# Therapeutic Challenges Derived from the Interaction Among Apolipoprotein E, Cholesterol, and Amyloid in Alzheimer’s Disease

**DOI:** 10.3390/ijms252212029

**Published:** 2024-11-08

**Authors:** Manuel Menendez-Gonzalez

**Affiliations:** 1 Departamento de Medicina, Facultad de Ciencias de la Salud, Universidad de Oviedo, ES-33006 Oviedo, Spain; menendezgmanuel@uniovi.es; 2 Servicio de Neurología, Hospital Universitario Central de Asturias, ES-33011 Oviedo, Spain; 3 Instituto de Investigación Sanitaria del Principado de Asturias (ISPA), ES-33011 Oviedo, Spain

**Keywords:** cholesterol, apolipoprotein, amyloid, Alzheimer’s, statins, immunotherapy

## Abstract

The isoform E4 of the Apolipoprotein E (ApoE) represents one of the strongest genetic risk factors for late-onset Alzheimer’s disease (AD). ApoE has key roles in cholesterol transport and amyloid-β (Aβ) metabolism, which are both central to AD pathogenesis. The E4 isoform has been implicated in reduced cholesterol homeostasis, increased Aβ aggregation, and heightened tau phosphorylation, contributing to amyloid plaques and neurodegeneration. This manuscript examines the complex interactions among ApoE isoforms, cholesterol metabolism, and amyloid pathology. Moreover, the therapeutic challenges associated with lipid-lowering agents (e.g., statins, PCSK9 inhibitors), anti-amyloid immunotherapies, and anticoagulants are described, focusing on ApoE4 carriers. Decision-making challenges are discussed by analyzing the pros and cons of these therapies.

## 1. Introduction

Alzheimer’s disease (AD) is the leading cause of dementia worldwide and is characterized by progressive memory loss, cognitive dysfunction, and ultimately, neurodegeneration. Two pathological hallmarks define AD: the accumulation of extracellular amyloid-β (Aβ) plaques and the intracellular formation of neurofibrillary tangles (NFTs) composed of hyperphosphorylated tau protein [1]. The cause of sporadic AD is multifactorial, involving genetic, environmental, and lifestyle factors. Among genetic factors, the E4 allele of Apolipoprotein E (ApoE4) stands out as the most significant [2,3,4]. ApoE is the principal lipid and cholesterol transporter in the central nervous system (CNS). Neuroinflammation has emerged as the third hallmark of AD, alongside amyloid-β (Aβ) plaques and neurofibrillary tangles composed of hyperphosphorylated tau.

This paper explores the molecular mechanisms by which ApoE, particularly ApoE4, influences cholesterol transport, Aβ metabolism, and AD pathogenesis. It further delves into the therapeutic challenges faced by ApoE4 carriers, with an emphasis on lipid-lowering therapies and anti-amyloid immunotherapies. The importance of personalized medicine and decision-making tools in managing these patients is underscored today.

## 2. ApoE and Cholesterol Metabolism: Influence on Alzheimer’s Disease, Vascular Health, and Amyloid Angiopathy

ApoE plays a crucial role in lipid metabolism, particularly in the transport and clearance of cholesterol and other lipids in the brain. Astrocytes and microglia synthesize ApoE in the central nervous system (CNS) and aid in maintaining neuronal membrane integrity and synaptic plasticity [5]. The different isoforms of ApoE (ApoE2, ApoE3, and ApoE4) have varying effects on AD risk. ApoE4, in particular, is associated with reduced Aβ clearance, increased amyloid deposition, and exacerbated neuroinflammation [3,4]. Furthermore, ApoE4 carriers are at higher risk of developing cerebral amyloid angiopathy (CAA), which is a condition characterized by amyloid deposition in cerebral blood vessels, increasing the risk of intracerebral hemorrhage (ICH) [5,6,7,8]. Moreover, ApoE4 has been shown to upregulate inflammatory pathways, particularly through the activation of microglia, leading to chronic neuroinflammation [9]. This sustained inflammatory response exacerbates amyloid pathology and contributes to neurodegeneration. In contrast, ApoE2 exhibits protective effects against amyloid deposition. ApoE2 has a higher affinity for Aβ and facilitates its clearance, thereby reducing amyloid burden. Additionally, ApoE2 is associated with decreased inflammatory responses, further highlighting its potential therapeutic implications. These insights into the contrasting roles of ApoE isoforms suggest that targeted strategies modulating ApoE2 levels could be explored as potential therapeutic avenues.

Cholesterol is involved in the processing of APP, which is the precursor to Aβ. APP is cleaved by β-secretase and γ-secretase, which are enzymes that reside in cholesterol-rich lipid rafts within neuronal membranes [8]. High cholesterol levels have been shown to play a pivotal role in the amyloidogenic processing of APP. Cholesterol enhances the activity of β-secretase (BACE1), leading to an increase in the amyloidogenic cleavage of APP and subsequent Aβ production. Furthermore, high cholesterol decreases α-secretase activity, thus diminishing the non-amyloidogenic pathway. Cholesterol also promotes the co-localization of APP and secretases in lipid rafts, which are lipid-rich microdomains in the neuronal membrane, in which amyloidogenic cleavage is favored. In addition to enhancing Aβ formation, cholesterol contributes to Aβ aggregation and impairs its clearance, thus exacerbating amyloid plaque formation. Cholesterol metabolism is also closely linked to tau pathology. High cholesterol levels have been shown to influence tau hyperphosphorylation through the activation of kinases such as glycogen synthase kinase 3β (GSK3β). Additionally, alterations in cholesterol metabolism can disrupt membrane integrity and intracellular signaling, contributing to the spread of tau pathology. These findings underscore the importance of considering cholesterol-modifying strategies in addressing both amyloid and tau pathologies.

Cholesterol metabolism is tightly regulated by ApoE, particularly in ApoE4 carriers, which show increased cholesterol deposition in lipid rafts and a higher propensity for amyloid deposition in both parenchymal and vascular compartments. Aβ and ApoE are predominantly produced by neurons and astrocytes, respectively. ApoE regulates Aβ oligomerization, aggregation, and receptor-mediated clearance, contributing to Aß pathology in AD. These molecular interactions underscore the interplay between cholesterol and amyloid pathology in ApoE4 carriers, indicating potential therapeutic targets. In ApoE4 carriers, dysregulated cholesterol metabolism leads to the accumulation of cholesterol in these lipid rafts, enhancing the amyloidogenic cleavage of APP and increasing Aβ production [8]. This contributes to the accumulation of amyloid plaques, which are a key feature of AD [10].

ApoE4 carriers are prone to arterial amyloidosis, in which amyloid deposits accumulate within the walls of cerebral arteries. This process is particularly concerning in individuals with CAA, as the amyloid-laden vessels become fragile and prone to rupture [11,12,13,14,15,16,17,18,19,20]. ApoE4 and ApoE2 promote the deposition of Aβ40 in the vasculature, leading to reduced vascular reactivity and increased susceptibility to brain hemorrhage [11,12,13,14,15,16,21]. Thus, arterial amyloidosis represents a significant concern in managing ApoE4 carriers, particularly when considering therapies that could exacerbate vascular fragility, such as anti-Aβ immunotherapies, anticoagulants, and statins [17,18,19,20].

## 3. Therapeutic Challenges and Decision-Making in the Management of ApoE4 Carriers

Managing ApoE4 carriers presents unique therapeutic challenges, particularly in balancing cardiovascular benefits against cerebrovascular risks. ApoE4 affects lipid metabolism, Aβ clearance, and tau pathology, all contributing to more complex clinical presentations in AD patients.

Statins reduce low-density lipoprotein cholesterol (LDL-C) levels by inhibiting HMG-CoA reductase. Statins are commonly prescribed to lower cholesterol levels and reduce the risk of cardiovascular disease. Statins are a class of drugs that primarily work by inhibiting the enzyme HMG-CoA reductase, which is crucial for cholesterol synthesis. Various statins, including simvastatin, atorvastatin, and rosuvastatin, are commonly used for lipid-lowering in cardiovascular diseases and AD. Understanding the pharmacokinetics and differential effects of these statins can inform therapeutic choices for AD management [22]. Simvastatin is lipophilic and can cross the blood–brain barrier, potentially exerting direct effects on brain cholesterol metabolism. Atorvastatin, also lipophilic, has been associated with neuroprotective effects due to its ability to modulate inflammatory pathways. Rosuvastatin, being hydrophilic, has limited penetration into the brain but is effective in lowering peripheral cholesterol levels (Table 1). Ezetimibe, a cholesterol absorption inhibitor that targets uptake at the jejunal enterocyte brush border, can be used alternatively or in combination with statins, while PCSK9 inhibitors such as Evolocumab and Alirocumab, are indicated in familiar hypercholesterolemia (Table 1).

While statins can reduce low-density lipoprotein cholesterol (LDL-C) levels and improve vascular health, their effects on AD progression are complex. Early interventions with statins may help mitigate the risk of Aβ accumulation by modulating cholesterol metabolism and enhancing clearance pathways. However, statin efficacy in modifying AD pathology may diminish as the disease progresses, due to established amyloid and tau pathologies. Several clinical trials have investigated the association between statin use, AD risk, and cognitive decline [23,24]. Findings from studies are heterogeneous, with variations in statin types, dosages, and populations studied. Overall, the evidence suggests that statins may have differential effects based on ApoE status and baseline cholesterol levels, indicating a need for personalized approaches. Indeed, recently, statins were shown to reduce the risk of AD in ApoE4 carriers [25]. However, their use in ApoE4 carriers has raised concerns due to the increased risk of hemorrhagic stroke, although the groups of patients in whom the risk of hemorrhagic stroke increases remain controversial [26,27,28,29]. One of these groups is individuals with CAA, which is more prevalent in AD with ApoE4.

Thus, in ApoE4 carriers, the decision to use statins must carefully weigh the benefits of cardiovascular protection against the increased risk of intracerebral hemorrhage. Several studies have shown that statins are associated with a reduced risk of ischemic stroke; however, they may also increase the risk of hemorrhagic stroke, particularly in individuals with CAA. Nonetheless, these findings are considered to lack sufficient robustness and highlight the need for additional focused research [30]. Before initiating statin therapy, clinicians should assess the patient’s cerebrovascular status using MRI to evaluate for microbleeds and apply the Boston criteria for diagnosing CAA [28,29]. If CAA is present, or in ApoE4 carriers, PCSK9 inhibitors and Ezetimibe offer an alternative lipid-lowering strategy. Particularly, PCSK9 inhibitors work by reducing the degradation of LDL receptors, thereby increasing the clearance of LDL cholesterol from the bloodstream. These agents have been shown to significantly lower LDL-C levels without increasing the risk of hemorrhagic stroke, making them a viable option for ApoE4 carriers or in any subject with a high CAA burden [31].

Anti-amyloid mAbs approved by the FDA for the treatment of AD, like Aducanumab, Lecanemab, and Donanemab have been developed to reduce amyloid plaques in the brains of AD patients by targeting Aβ plaques and facilitating their clearance through immune-mediated mechanisms. Aducanumab, Lecanemab, and Donanemab are anti-amyloid monoclonal antibodies that target different forms of Aβ. Aducanumab primarily targets aggregated Aβ plaques, facilitating their clearance via microglial activation. Lecanemab binds to Aβ protofibrils, reducing the formation of larger aggregates and promoting clearance. Donanemab selectively targets modified Aβ plaques, resulting in plaque reduction.

However, this mechanism of action can induce inflammation in brain tissues and blood vessels, leading to amyloid-related imaging abnormalities (ARIA). Specifically, the activation of microglia and other immune cells may cause increased permeability of the blood–brain barrier, leading to microhemorrhages that can progress to larger hemorrhagic events. This risk is notably higher in ApoE4 carriers, who have more severe amyloid deposition in cerebral vessels, predisposing them to CAA. The risk of hemorrhagic stroke under anti-amyloid mAb therapy is often dose-dependent and related to the duration of treatment. Thus, understanding the specific risk factors and careful monitoring of ApoE4 carriers are essential for optimizing the safety of these therapies. However, the use of these therapies in ApoE4 carriers is associated with an increased risk of ARIA, particularly ARIA-H (microhemorrhages) and ARIA-E (cerebral edema) [32,33,34,35,36,37,38]. Thus, ApoE4 homozygous carriers are at significantly higher risk for ARIA when treated with these therapies, which raises important concerns about their safety in this population. Alternative therapies or alternative routes of delivery (i.e., intrathecal pseudodelivery) should be considered in these patients [39]. In this route, mAbs are administered after being dissolved in a solution and contained within a subcutaneous capsule fitted with a nanoporous membrane that is connected to the spinal CSF with an intrathecal catheter. The nanoporous membrane prevents the mAbs from escaping while permitting target molecules, such as soluble Aβ, to enter the capsule. Once inside, the mAbs interact with Aβ, capturing and removing them from the CSF. This mechanism of action is expected to increase efficacy at the time of avoiding immunotherapy-induced ARIA. 

Given the complexity of managing ApoE4 carriers, shared decision-making with patients and their families is essential. The high risks associated with both anti-amyloid therapies and anticoagulants should be communicated, and treatment decisions should align with the patient’s values and preferences. Involving patients in the decision-making process ensures that they are informed about treatment’s potential risks and benefits and that their health goals are prioritized. This goes beyond statins or immunotherapies; for instance, anticoagulation therapy is often indicated in patients with atrial fibrillation to reduce the risk of ischemic stroke. However, ApoE4 carriers with CAA face a heightened risk of hemorrhagic stroke when treated with anticoagulants. Tools such as the ICH score and CHA2DS2-VASc score can help guide decision-making, but in patients with significant amyloid burden, the risk of ICH may outweigh the benefits of stroke prevention.

Expert guidelines emphasize the need for patient selection based on genetic risk factors and baseline neuroimaging and suggest avoiding treatment in patients with more than four microhemorrhages or significant white matter disease, as these conditions increase the likelihood of severe complications [40,41,42]. Additionally, anticoagulant use is strongly discouraged in APOE4 carriers undergoing anti-amyloid therapy due to the heightened risk of macrohemorrhage. In cases in which anticoagulation is necessary, switching to antiplatelet therapy might be a safer option [40,41,42]. Tools such as the ICH score and CHA2DS2-VASc score can help guide decision-making, but in patients with significant amyloid burden, the risk of ICH may outweigh the benefits of stroke prevention. Intriguingly, a long-used anticoagulant drug, heparin, may delay the onset of AD by approximately one year [43]. While heparin is unlikely to be prescribed for AD, it reinforces the idea that naturally occurring brain chemicals, like heparan sulfate proteoglycans (HSPGs), are involved in the disease. HSPGs bind key AD-related proteins, ApoE and tau, with ApoE4, the strongest genetic risk factor for late-onset AD, showing a higher affinity for HSPGs. This study analyzed electronic health records from two independent hospital systems and found that past heparin use was associated with a later AD diagnosis compared to those who did not receive the drug. The hypothesis is that heparin may compete with HSPGs for binding sites on ApoE and tau, thereby reducing AD pathology. The protective effect persisted even after adjusting for comorbidities, which could otherwise confound the results. These findings suggest that further research into AD treatments mimicking heparin’s mechanisms might be beneficial and highlight the power of combining large-scale health data with biologically informed hypotheses.

ApoE4 can be a target itself in AD, which is being explored under the following different therapeutic strategies (Table 2) [44,45,46]:

1. Reduction of ApoE Levels: Since ApoE4 exerts toxic gain-of-function effects, one promising approach is to reduce its levels. The administration of ApoE-specific antisense oligonucleotides (ASOs) and small interfering RNAs (siRNAs) has shown notable pharmacological effects in preclinical studies. These therapies work by decreasing ApoE expression, which, in turn, leads to significant reductions in amyloid-β (Aβ) deposition, neuroinflammation, and synaptic dysfunction in transgenic mouse models. Such results underscore the potential of ApoE-targeted therapies to alleviate AD pathologies and improve cognitive outcomes.
2. Structural and Functional Stabilization: Another avenue being explored is the structural correction of the ApoE4 protein. ApoE4’s distinct conformation leads to its instability and propensity to form toxic fragments. Small molecules or peptides that stabilize ApoE4 or modulate its folding toward a less pathogenic conformation are under investigation. These interventions aim to neutralize the harmful effects of ApoE4 on neurons, synaptic integrity, and blood–brain barrier function.
3. Modulation of Lipid Transport: ApoE4 has altered lipid-binding properties that disrupt cholesterol homeostasis in the brain. As cholesterol plays a key role in maintaining neuronal membrane integrity and synaptic plasticity, therapeutic strategies aiming to restore normal lipid transport by modifying ApoE4-lipid interactions could help alleviate downstream effects, such as amyloid-β aggregation and tau hyperphosphorylation.
4. Gene Editing Approaches: Recent advancements in gene editing technologies, such as CRISPR/Cas9, have opened the possibility of directly modifying the APOE gene to either silence the E4 allele or replace it with a protective variant, such as APOE2. Although still in experimental stages, this approach offers a potential long-term solution to mitigate the genetic risk posed by ApoE4. Given the diverse mechanisms by which ApoE4 influences AD pathogenesis—ranging from impaired amyloid clearance to increased neuroinflammation—targeting ApoE4 represents a comprehensive strategy to address the disease at multiple levels. The growing body of evidence supporting the efficacy of ApoE-lowering therapies and structural modulation provides a strong rationale for advancing these approaches into clinical trials. A recent gene therapy trial has reported promising results in targeting the underlying cause of APOE4-associated AD. This innovative therapy, the first of its kind, aims to address the root genetic factors linked to the condition. Findings from the Phase 1/2 trial indicate a dose-dependent increase in APOE2 levels, accompanied by improvements in biomarkers associated with cognitive health. The therapy employs an AAV-based delivery system to achieve these results [44,45,46].

In summary, targeting ApoE4 in AD is a multifaceted approach, involving the reduction of ApoE levels, stabilization of its structure, modulation of lipid transport, and even gene editing techniques through a variety of mechanisms of action and routes of delivery. These strategies offer promising therapeutic avenues for addressing the underlying pathology of AD in patients carrying the APOE ε4 allele that are currently in different stages of development (Table 2).

With all of these therapeutic strategies, the need for continuously updated clinical guidelines addressing the management of ApoE4 carriers with cardiovascular and cerebrovascular comorbidities is urgent. While current therapies offer significant benefits, their risks must be carefully balanced, particularly in high-risk populations like ApoE4 carriers. Future research should focus on developing safer and more effective lipid-lowering and anti-amyloid therapies tailored to the specific needs of ApoE4 carriers.

## 4. Conclusions

Clinicians must carefully balance the benefits of statins and anti-amyloid therapies with the potential for hemorrhagic complications in this genetically at-risk population.

Before initiating statin therapy or any anti-amyloid therapies, clinicians should test ApoE polymorphism to check if ApoE4 or CAA is present in order to balance risks and benefits.

In ApoE4 carriers or any patient with CAA, alternative lipid-lowering strategies, such as Ezetimibe and PCSK9 inhibitors, should be considered, and anti-amyloid therapies should be avoided or used with extreme caution. Alternative routes of delivery for anti-amyloid mAbs, such as intrathecal pseudodelivery, might be a feasible option in the future.

ApoE4 can be a target itself in AD. Different therapeutics strategies are being developed that will offer alternative treatment options to this group of patients.

## Figures and Tables

**Table 1:** Statins and PCSK9 inhibitors for cholesterol management.

Medication	Mechanism of Action	Key Side Effects
Atorvastatin	Inhibits HMG-CoA reductase	Myopathy, increased liver enzymes, risk of hemorrhagic stroke
Simvastatin	Inhibits HMG-CoA reductase	Myopathy, rhabdomyolysis, cognitive impairment
Rosuvastatin	Inhibits HMG-CoA reductase	Myalgia, liver enzyme elevation
Evolocumab	PCSK9 inhibitor	Nasopharyngitis, myalgia, injection site reactions
Alirocumab	PCSK9 inhibitor	Nasopharyngitis, myalgia, skin reactions
Ezetimibe	Cholesterol absorption inhibitor	Gastrointestinal

**Table 2:** Therapeutic strategies targeting ApoE4 in AD, along with the route of delivery and current development stage.

Therapeutic Strategy	Mechanism of Action	Route of Delivery	Current Development Stage
ApoE Antisense Oligonucleotides (ASOs)	Reduces ApoE expression to decrease toxic effects of ApoE4, lowering amyloid-β deposition and neuroinflammation.	Intrathecal or intravenous injection	Preclinical and early clinical studies
ApoE siRNAs	Interferes with ApoE mRNA to decrease ApoE protein levels, reducing amyloid deposition and neuroinflammation.	Intrathecal or intravenous injection	Preclinical and early clinical studies
ApoE Structural Stabilizers	Stabilizes the ApoE4 protein structure to prevent formation of toxic fragments and improve neuronal health.	Oral or intravenous administration	Preclinical
Lipid Modulation Therapy	Restores normal lipid transport to maintain neuronal membrane integrity and reduce amyloid aggregation.	Oral or intravenous administration	Preclinical
Gene Editing (CRISPR/Cas9)	Directly modifies the APOE gene to silence or replace the E4 allele with a protective variant (e.g., APOE2).	Intracerebral or systemic delivery	Preclinical
